# Phytochemical Profiling and Anti‐Bacterial Activity of Red Delicious Apple Pomace With Integrated Hydrolysis to Obtain Fermentable Sugars: A Biorefinery Approach

**DOI:** 10.1002/fsn3.70543

**Published:** 2025-07-09

**Authors:** Nisha Devi, Natália Cruz‐Martins, Tabarak Malik, Vinod Kumar, Muhammad Torequl Islam, Rachna Verma, Gurvendra Pal Singh, Dinesh Kumar

**Affiliations:** ^1^ School of Bioengineering and Food Technology Shoolini University of Biotechnology and Management Sciences Solan Himachal Pradesh India; ^2^ Life and Health Sciences Research Institute (ICVS), School of Medicine University of Minho Braga Portugal; ^3^ Faculty of Medicine University of Porto Porto Portugal; ^4^ Department of Biomedical Sciences, Institute of Health Jimma University Jimma Ethiopia; ^5^ Division of Research and Development Lovely Professional University Punjab India; ^6^ School of Water, Energy and Environment Cranfield University Cranfield UK; ^7^ Department of Pharmacy Bangabandhu Sheikh Mujibur Rahman Science and Technology University Gopalganj Bangladesh; ^8^ School of Biological and Environmental Sciences Shoolini University of Biotechnology and Management Sciences Solan Himachal Pradesh India; ^9^ Centre of Advanced Innovation Technologies VSB‐Technical University of Ostrava Ostrava Czech Republic

**Keywords:** antioxidant, apple pomace, biorefinery, enzymatic hydrolysis, polyphenols

## Abstract

Apple pomace (AP) is a byproduct of juice processing, rich in nutritionally important compounds like carbohydrates, phenolic compounds, dietary fiber, and minerals. It is a potential feedstock for sugar‐based biorefineries. This study explored AP physicochemical and bioactive compounds and their hydrolysis to extract fermentable sugar. Results showed that AP had a below‐neutral pH, moisture, acidity, and ash. The AP contained crude fiber (27.22%), total sugar (36.75%), and reduced sugar (12.22%). The extracts contained minerals like potassium, calcium, magnesium, aluminum, iron, boron, and zinc. A GC–MS study analyzed the phytochemicals present in AP extracts, revealing prominent antibacterial activity. The optimal conditions for enzymatic hydrolysis were found to be 1 mg/g substrate of cellulase and pectinase at 50°C and pH 5.0 at 24 h of incubation. Enzymatically hydrolyzed AP showed a high yield of reducing sugar (38.33%) compared to non‐hydrolyzed AP (12.22%). The study suggests that AP, currently discarded as industrial bio‐waste, is still a source of phytochemicals with significant antioxidant and antibacterial activities.

## Graphical abstract

The study explores the use of integrated hydrolysis in the biorefinery approach to analyze the phytochemical profile and anti‐bacterial activity of red delicious apple pomace and its applications in biorefinery.

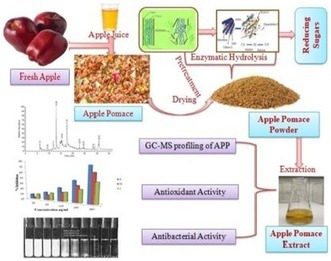

## 1 Introduction

The apple processing industry primarily generates apple pomace (AP) as its main byproduct. It is mainly composed of the skin, seeds, and stems that remain after the juice extraction from the fruit. AP is a solid leftover that is generated after the separation of apple juice and comprises about 25%–30% of the total processed apples (Zaky et al. 2024). Every day, millions of tons of AP are produced throughout the world. AP comprises a wide range of phytochemicals, including simple sugars, pectin, dietary fibers, natural antioxidants, and essential nutrients (Bhushan et al. 2008). Among its many constituents are insoluble polysaccharides. AP is mostly composed of cellulose, hemicellulose, lignin, and simple sugars, which are insoluble carbohydrates (Kauser et al. 2024).

Fresh AP contains cellulose (7%), protein (5%), water (70%), carbohydrates (16%), and a variety of important phytonutrients with antioxidant properties (Zaky et al. 2024; Kruczek et al. 2016). The average annual production of AP worldwide is predicted to be 4 million tons, and future growth is anticipated. In general, the retrieval rate for AP is notably limited and insufficient. Directly dumping of AP into the ground in a landfill is the disposal technique that is used most frequently. Because AP contains a lot of water (> 70%), soluble and insoluble sugars, and natural acids and all are prone to fast microbial fermentation and adversely harm the land and water. The increase in the microbial flora may affect the C/N ratio, resulting in less available nitrogen in the soil (Lyu et al. 2020).

To reduce the risk of environmental and health hazards, AP must be treated and used effectively and safely. Commercial AP applications have the potential to have significant economic effects in the future due to the enormous volume of AP produced (Płotka‐Wasylka et al. 2017). AP has numerous applications nowadays, such as composting, pectin extraction, and integration into feed and food systems (Awasthi et al. 2021).

AP may also be used to produce environmentally friendly biological polymers (Liu et al. 2021). AP is considered an important raw material that can be processed or reused to promote sustainability. The recovery and use of AP, which has the potential to be reused and reintroduced into the supply system, are important for its sustainable management strategy. Green extraction methods enable the environmentally friendly production of AP extracts that contain active ingredients. The solid waste products produced during extraction can be stabilized and further processed into waste‐free biopolymers or alternative energy sources. As a result, there is a possibility for decreasing environmental pollution and improving waste integration into a circular economy, which may lead to sustainable AP management (Campos et al. 2020).

An analysis of food waste management alternatives reveals that the first option for apple waste is to make it fit for human consumption by enhancing the nutritional content of baked goods (Tulej and Głowacki 2022). Many studies have been done on using AP to produce bioethanol, butanol, 2,3‐butanediol, and organic acids such as citric, fumaric, propionic, lactic, and acetic acids (Costa et al. 2022).

According to Tulej and Głowacki (2022), in biorefining processes that maximize substrate conversion, energy consumption, and chemical consumption, AP may be a good substrate. AP is more appealing and financially feasible when valued as part of the biorefinery strategy than when used as a stand‐alone product source. Biorefineries have a great deal of promise for producing biofuels and organic fertilizers from biomass waste (Awasthi et al. 2021).

Since AP is currently discarded as industrial bio‐waste, the current study aims to investigate its proximate analysis, phytochemical composition, and bioactivity to confirm its prospect as a novel resource of biorefinery to formulate value‐added products and hydrolysis of AP for the extraction of soluble and non‐soluble‐sugars‐for‐further‐applications‐in‐biorefinery and used as a promising feedstock for the production of itaconic acid which is used as a substitute for many petroleum‐based compounds that can help in promoting the environmental sustainability and circular economy.

## 2 Materials and Methods

### 2.1 Sample Preparation

The Royal Delicious variety of red apples was procured from the market and identified at the Department of Fruit Science, Dr. Y.S. Parmar University of Horticulture and Forestry, Solan, Himachal Pradesh, India, with reference No. UHF/FS/Test/2021/2714. All apples were thoroughly cleaned with a potassium permanganate solution (0.1%) and washed with tap water. Sliced apples were blanched with sodium hypochlorite solution (50–200 mg/L of water) for 15 min, crushed in a fruit crusher, and apple juice was removed through a muslin cloth. AP was dried in a hot air oven at 60°C for 48 h before being crushed into a powder and kept at 20°C in an airtight container.

### 2.2 Chemical Characterization of Apple Pomace

#### 2.2.1 Proximate Analysis of Apple Pomace Powder

AOAC (2005) methods were used to determine the proximate composition of AP powder, which included moisture, pH, titratable acidity, crude protein, crude fat, ash, TSS (total soluble solids), total reducing sugar, and crude fiber. Protein was estimated through the Kjeldahl distillation method and calculated by multiplying the amount of nitrogen by 6.25. 10 g of the sample was weighed in a Kjeldahl flask, catalyst mixture (10 parts K_2_SO_4_ to one part of CuSO_4_), and 25 mL of concentrated H_2_SO_4_ were added. The ash content of the flask was digested under boiling at maximum heat for 2–3 h till clear, and then the flask was distilled using NaOH 40%, the ammonia was received in a 100 mL conical flask containing 10 mL of 0.1 N HCl. Inductively Coupled Plasma‐Optical Emission spectroscopy (ICP‐OES) was used for the estimation of mineral content.

#### 2.2.2 Determination of Phytochemicals of Apple Pomace

##### 2.2.2.1 Total Phenolic Content

The total phenolic content (TPC) of methanol and chloroform extracts of AP was estimated using the folin–ciocalteu reagent method (Senguttuvan et al. 2014; Aregbe et al. 2024). A 20 μg of AP extract was taken and the volume was made to 1 mL with distilled water. Then, 500 μL of the FC reagent and 2.5 mL of 20% Na_2_CO_3_ were added. The absorbance/concentration of the sample extract was measured at 725 nm using a UV–Vis Spectrophotometer (Thermo Fisher, Evolution 201, UV–visible spectrophotometer, USA). A standard calibration curve was prepared using gallic acids in the range of 2.5–20 μg/mL. The TPC of AP extracts was calculated in milligrams of gallic acid equivalent (GAE)/100 g of the sample using a calibration curve.

##### 2.2.2.2 Total Flavonoids Content

For the estimation of flavonoids present in AP, 250 μL of AP extract was taken and added to 4500 μL of distilled water, and then 300 μL of NaNO_2_ (5%) was added. Then 300 μL of AlCl_3_ (10%) was added and incubated for 6 min and after incubation, 2000 μL of NaOH (1 M) was added and a volume was made up to 10 mL with distilled water. After this, the sample mixture was agitated, and the concentration of the sample was measured at 510 nm and calculated as rutin equivalents (Senguttuvan et al. 2014; Aregbe et al. 2024).

##### 2.2.2.3 Tannin Content Estimation

In the estimation of tannins, about 0.1 mL of the AP extract was taken into a test tube and added to 500 μL of Folin–Ciocalteu solution, followed by the addition of 1000 μL of 35% Na_2_CO_3_, and the final volume was made up to 10 mL with distilled water. The solution was vortexed and incubated for 30 min at 25°C. Different concentrations of tannic acid (20–100 μg/mL) were used for standard calibration curve preparation. A UV/visible spectrophotometer was used to quantify the concentrations of the test sample and standard against their optical density at 700 nm. The concentration of tannin content was measured as mg/100 g of tannic acid in the AP (CI and Indira 2016).

##### 2.2.2.4 Alkaloid Content Estimation

The amount of crude alkaloid in AP powder (APP) was measured by gravimetric method (Yadav and Gupta 2015). For this, 2.5 g of dried APP (W_1_) was taken in a conical flask and dissolved in 100 mL (10%) acetic acid (prepared in ethanol), then kept at 25°C for 4 h. After incubation, the sample was filtered with Whatman filter paper No. 1 and then resolute to one‐fourth of the initial volume at 50°C in a water bath. Then, concentrated liquid ammonia (35%) was added to the concentrate drop by drop until precipitates formed. Again, the sample was filtered with pre‐weighed (W_2_) Whatman filter paper No. 1. The oven‐dried filter paper was weighed again (W_3_) and alkaloid content was measured by the following equation:

$$\%\text{alkaloid content}=\frac{\text{Final weight}\left(W_{3}\right)–\text{Initial weight}\left(W_{2}\right)}{\;\text{Sample weight}\left(\;W_{1}\right)}\;X100$$

##### 2.2.2.5 Estimation of Saponin Content

The saponin content of APP was measured according to the method described by Rahman et al. (2013) with some modifications. A 5 g sample of APP was dissolved in 50 mL of 20% ethanol, and the mixture was placed in a water bath at 55°C for 4 h with constant stirring. The mixture was passed through filters and reduced to 10 mL in a water bath at 90°C. In a separating funnel with constant manual shaking, diethyl ether was added to this concentrate mixture. The aqueous layer was separated, and then n‐butanol was added. After this, the solution obtained was rinsed with 10 mL of aqueous NaCl (5%) solution. The solution obtained was boiled in a water bath. Then the resulting sample was oven‐dried until it reached a consistent weight, and % saponin content was calculated as follows:

$$\%\text{saponins content}=\frac{\text{Weight of saponins}}{\text{Sample weight}}\times 100$$

#### 2.2.3 GCMS/MS Analysis

The samples of methanol and chloroform extracts of AP were prepared for GCMS analysis with the same solvents in a ratio of 1 mg/mL. GC–MS was used to examine the fraction components (GC‐Trace1300/GCMS‐TSQ‐Duo, Thermo Fisher, USA), prepared with an auto‐sampler (TriPlusRSH) and TG‐5MS column (40 m × 0:15 mm × 0.15 μm). The operating conditions of GC–MS as stated by Al‐Owaisi et al. (2014) and Göncü (2024) were used to screen AP extracts with minor adjustments. The starting temperature of the process was set at 70°C and maintained for 1 min. Following that, the temperature was raised at a rate of 7°C per minute until it reached 270°C and maintained for 2 min. Finally, the temperature was at 270°C and was sustained for 20 min. The temperature of the transfer line was 250°C; the carrier gas was helium at a constant flow rate of 0.7 mL/min with the splitless injection mode; the component ionization mode was electron impact (70 eV); the ion source temperature was 250°C; the run time was 50 min; and the mass range was 45–450 m/z.

#### 2.2.4 Test for Antioxidant Activity of Apple Pomace

##### 2.2.4.1 Radical Scavenging Activity (DPPH Assay)

Senguttuvan et al. (2014), method was used for determining the scavenging capacity of free radicals in AP methanol and chloroform extracts with slight modifications. Solutions of fixed concentrations of both extracts (1 mg/mL) in methanol were made, and a series of methanol‐soluble extract concentrations (30, 60, 120, 240, and 480 μg/mL) were prepared. To measure the absorbance, 0.3 mL of solution was taken from each concentration and added to 2700 μL of DPPH (4 mg of 2,2‐diphenyl‐1‐picrylhydrazyl) dissolved in 100 mL of methanol, prepared fresh every time. The solution mixer was incubated at 37°C for 1 h. The optical density or absorbance of the sample solution was measured at 517 nm. Ascorbic acid was used for the standard curve preparation as a positive control (Senguttuvan et al. 2014; Fatemi et al. 2024). The radical scavenging ability in the percentage of DPPH was calculated using the following formula:

$$\%\text{DPPH}\;\left(\text{radical scavenging ability}\right)=\frac{A_{\text{Blank}-}\;A_{\text{Sample}}}{A_{\text{Blank}}}\times 100$$

The linear curve regression equation of residual DPPH absorbance versus concentration of the sample was used to calculate the half‐minimum inhibitory concentration (IC_50_) of DPPH radical.

##### 2.2.4.2 FRAP Assay

The ferric‐reducing antioxidant capacity of AP was determined using the method described by Benzie and Strain (1996) and Altemimi et al. (2023) with a few modifications. FRAP reagent was freshly prepared by incorporating 2.5 mL of 2,4,6‐tripyridyl‐S‐triazine (10 mM TPTZ in 40 mM hydrochloric acid), 2.5 mL (20 mM) FeCl_3_, and 25 mL (300 mM) sodium acetate buffer (pH 3.6). A 0.1 mL of AP extract was taken, and 0.9 mL of FRAP reagent was mixed into it. The optical density of unknown samples was measured at 593 nm, and the concentration was calculated with a linearity curve of ferrous sulfate and expressed as ferrous sulfate equivalents.

#### 2.2.5 Antibacterial Sensitivity Assay

The minimum inhibitory concentration (MIC) of the AP was determined by the macrodilution method (Andrews 2001), with some modifications. Three gram‐negative bacteria and two gram‐positive bacteria viz. *Salmonella typhimurium* (MTCC 3858), *K. pneumonia* (MTCC 109), *Escherichia coli* (MTCC 443), *Bacillus cereus* (MTCC 1305), and *Enterococcus faecalis* (MTCC 1032) were used to check the antibacterial potential of AP methanol and chloroform extracts (Altemimi et al. 2023). Methanol and chloroform extracts of AP were made in MHB (Mueller‐Hinton broth), and using serial dilutions in the concentration both the extracts were maintained between the range of 62.5 and 500 μg/mL. A bacterial suspension of 1 × 10^6^ CFU/mL was put in test tubes and incubated for 24 h at 37°C. The MIC was calculated as the lowest concentration when no apparent turbidity was detected. Ampicillin sodium salt was used as a control against bacteria (Andrews 2001).

### 2.3 Pre‐Treatment of Apple Pomace

#### 2.3.1 Hydrothermal Treatment

The hydrothermal treatment of AP was carried out with hot water extraction to obtain reducing or soluble sugars in AP (Jin et al. 2019). Dried AP powder (10 g) was moistened with distilled water (200 mL) in a ratio of 1:20 for the extraction of reducing sugar using a water bath at 100°C and autoclaving at 121°C for 30 min, respectively. Both samples (water bath and autoclaved) were centrifuged (10,000 rpm, 20 min, 4°C) to separate the liquid portion from the solid residue. The liquid part of the hydrothermally treated AP was used for the estimation of reducing sugar by using the 3,5‐dinitrosalicylic acid (DNS) method (Jin et al. 2019). The residual solid material was pretreated further and hydrolyzed enzymatically to produce soluble sugars.

#### 2.3.2 Alkaline Treatment

The solid residue from hydrothermal treatment of AP was subjected to pretreatment using sodium hydroxide (1% w/v) with a ratio of 1:20. This pretreatment of AP was optimized at different temperatures (100°C and 121°C) for 30 min to determine the most effective conditions. The liquid part containing soluble sugars was separated from the solid residual material by centrifugation for 20 min at 4°C at 10,000 rpm. The leftover residue was then rinsed 3–4 times with distilled water to remove all traces of NaOH and inhibitory components released during alkaline treatment. After this treatment, the treated sample was further utilized for the enzymatic hydrolysis (Jin et al. 2019).

#### 2.3.3 Enzymatic Hydrolysis of Pretreated AP

Pectinase and cellulase enzymes were used in the enzymatic hydrolysis of the above pretreated AP to extract hydrolyzed sugars. This treatment was carried out in two batches. In one batch, hydrothermally treated AP powder was treated with enzymes, and in another batch, the hydrothermally and alkaline‐treated AP was treated with the selected enzymes. The DNS method was used for calculating the reducing sugars released in both experiment setups. Sodium citrate buffer (0.1 M pH 5.0) was used for preparing the enzyme solution. Enzyme activity during the hydrolysis process was optimized with various factors that is, temperature (35°C–55°C), enzyme concentration (1%–5%), incubation time (0–48 h), and substrate concentration (1–5 g) (Lin et al. 2024). The most efficient conditions for all parameters, which yielded the maximum amount of reducing or soluble sugars while minimizing energy consumption, were recorded for further experiments. To collect the sugar hydrolysate from hydrolyzed AP samples, centrifugation was done at 4°C at 10,000 rpm for 20 min. The hydrolysate of the samples was used for the estimation of reducing sugars as discussed earlier (Jin et al. 2019).

### 2.4 Statistical Analysis

For each experiment, the findings are reported as the mean of three observations plus the standard deviation (*x* ± SD). The IC_50_ values were determined via nonlinear regression analysis of inhibition‐concentration curves. Microsoft Excel was used for the statistical analysis.

## 3 Results and Discussion

### 3.1 Nutritional and Chemical Profiling of Apple Pomace

The results of the proximate analysis of AP are presented in Table 1. The results of the physicochemical analysis showed that dried AP contains 12.22% total reducing sugars, while the insoluble sugar constituted 6.24% pectin (as calcium pectate) and 27.42% crude fiber. The crude protein and fat based on the dry weight of AP were 6.85% and 3.38%, respectively. The AP is composed of 36.75% total sugars, 81.63% total solids, and 4.87% ash in dry matter. Chandel et al. (2016) reported similar content of TSS (8.65°B), crude protein (6.12%), and ash (5.44%) and low content of total reducing sugar (6.25%), titratable acidity as malic acid (1.81%), and high content of ascorbic acid (19.30 mg/100 mg), pectin (14.66%), crude fiber (31.31%), and total sugar (38.05%) in AP. Yadav and Gupta (2015) also observed high moisture (6.6%), ash (11%), and crude fiber (30.86%) while low content of crude protein (4.63%) and fat (0.88%) in their study.

**TABLE 1:** Proximate composition and mineral profiling of apple (royal delicious) pomace obtained with ICP‐OES.

Proximate analysis		Mineral analysis	
Parameter	Composition (%)	Elements	Content (mg/kg)
Moisture	3.12 ± 0.13	Al	47.99 ± 0.17
pH	4.72 ± 0.19	B	13.85 ± 0.07
Ash	4.87 ± 0.66	Ba	7.50 ± 0.04
TSS (°Brix)	8.69 ± 0.24	Ca	4204.32 ± 3.57
Total solid	84.97 ± 0.57	Cu	4.86 ± 0.19
Titratable acidity (% malic acid)	1.48 ± 0.34	Fe	30.51 ± 1.32
Ascorbic acid	16.34 ± 0.28	K	7309.86 ± 4.89
Protein	6.85 ± 0.09	Mg	1967.18 ± 1.75
Crude fat	3.38 ± 0.15	Mn	8.33 ± 0.05
Reducing sugar	12.22 ± 2.00	Na	503.63 ± 0.25
Total sugar	36.75 ± 0.81	Ni	1.15 ± 0.04
Crude fiber	27.42 ± 0.54	Si	8.91 ± 0.11
Pectin (% calcium pectate)	6.24 ± 0.10	Zn	10.97 ± 0.16
Cellulose	10.47 ± 0.26		
Hemicellulose	26.18 ± 0.18		
Lignin	1.65 ± 0.35		
Alkaloid (%)	8.62 ± 0.03		
Saponin (%)	28.98 ± 0.34		

*Note:* Each value is presented as the mean *±* SD (*n* = 3).

In the present study, AP showed a lower amount of cellulose (10.47%), lignin (1.65%), and a higher concentration of hemicellulose (26.18%). However, Hijosa‐Valsero et al. (2017) reported 21.2% cellulose and 14.75% hemicellulose and higher concentrations of mineral content (1.31%) and lignin (18.5%) in AP as compared to the present study. These variations in chemical composition may be attributed to various apple cultivars or the technology utilized for biomass evaluations (Leonel et al. 2020).

Kruczek et al. (2023) reported that 4.27% protein, 2.03% fat, 12.3% soluble fiber 37.32% insoluble fiber (including cellulose, hemicellulose, and lignin), 0.87% ash, and 8.25% moisture.

The waste of apples was found to be a good source of sugars, pectin, acid, and minerals but low in crude proteins and fat. The AP is also used as a rich source of sugar and pectin for biorefinery applications in the synthesis of a few organic acids by value addition, which helps in its waste utilization and the circular economy (Fitria et al. 2023).

ICP‐OES was used to assess the mineral composition of the AP powder in the present study and revealed the presence of important elemental components such as Ca, Mg, Zn, Al, Fe, Cu, Mn, and many more, as shown in Table 1. The AP showed the highest amount of K (7309.86 ± 4.89) followed by Ca, Mg, and Na, respectively. Different minerals increase process efficiency and reduce costs while supporting the concept of a circular bioeconomy when used as a low‐cost feedstock in biorefineries (Yadav and Gupta 2015; Fitria et al. 2023). However, the concentration of minerals varied between geographical locations with apple orchards. This variation seems to result from differences in mineral availability in soils from different parts of the world (Kruczek et al. 2016).

### 3.2 Phytochemicals Study of AP

The AP extracts obtained with different solvents were studied for their phytochemicals, and the results are shown in Table 2. Phenolics and flavonoids play a vital role in the curing of diseases such as high blood pressure, cancer, and cardiovascular disease. Polyphenols in AP are mostly accountable for absorbing free radicals formed in the human body and are thus regarded as making a significant contribution to their antioxidant capacity (Senguttuvan et al. 2014).

**TABLE 2:** Phytochemical profile of apple pomace extracts.

Solvent extract of AP	Polyphenols (μg GAE/mg sample)	Flavonoids (μg RE/mg sample)	Tannins (μg TAE/mg sample)
Methanol extract	176.833 ± 0.001	55.763 ± 0.001	14.942 ± 0.001
Chloroform extract	57.286 ± 0.002	62.079 ± 0.001	11.877 ± 0.002

*Note:* All values are presented as mean *±* SD (*n* = 3).

Phenolics are antioxidants and secondary metabolites that contribute to nutritional and health benefits. Flavonoids are especially important phytochemicals that act as antioxidants (Barreira et al. 2019). Phytochemicals present in apple waste have also been shown to have few medicinal effects on Alzheimer's disease and diabetes, as well as in lowering postprandial blood glucose levels (Yadav and Gupta 2015; Rahman et al. 2013).

The maximum number of polyphenols was observed in methanol extract (176.833 μg/mg) followed by chloroform extract (57.286 μg/mg), while the flavonoid content of methanol extract (55.763 μg/mg) was observed less than the chloroform extracts (62.079 μg/mg). Previously, multiple studies had been carried out to find the TPC (12.74 μg/mg) and total flavonoids content (30.84 μg/mg) in industrial AP, and the findings revealed the greatest variation in dry AP. In addition, research has been conducted to optimize extraction conditions for phenolics in industrial AP utilizing the response surface approach (Adil et al. 2007; Rana et al. 2014).

According to the findings of Grispoldi et al. (2022) ultrasound‐assisted extraction (UAE) was a successful technique for extracting phenolic compounds, and UAE extract of AP showed TPC values of 8.56 mg GAE/g dry weight, while Lyu et al. (2020) reported phenolic acids content in AP was in the range 52.3–154.2 mg/100 g DM.

In the present study, saponins (28.98%) and alkaloids (8.62%) were also found in AP and showed that the tannins content of the methanol extract (14.942 μg/mg) was more than that of the chloroform extract (11.877 μg/mg) (Table 2). Yadav and Gupta (2015) also found similar amounts of saponins (28.40%) and alkaloids (4.38%) but less tannins content (0.0117 μg/mg) in their study.

### 3.3 Phytochemicals Analysis by GC–MS/MS–MS

**FIGURE 1:**
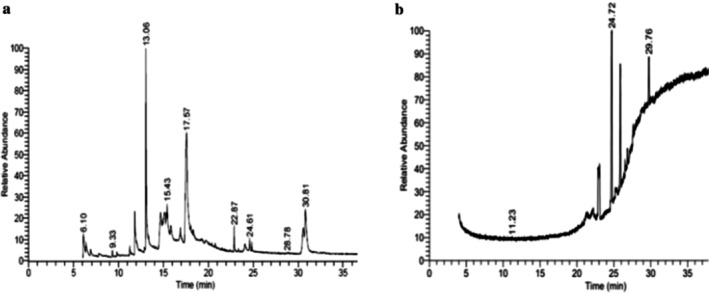
A GC–MS profile of (a) methanol extract and (b) chloroform extract of apple pomace.

The GC–MS/MS–MS profiling of methanol and chloroform extracts of AP was done for the recognition of bioactive compounds present in it (Figure 1a,b). The results obtained indicated that 19 compounds were identified in methanol extract and nine compounds in chloroform extract (Tables 3 and 4). The results showed that the 4H‐pyran‐4‐one, 2,3‐dihydro‐3,5‐dihydroxy‐6‐methyl; n‐hexadecanoic acid; octadecanoic acid; ç‐sitosterol; 2furancarboxaldehyde; 5(hydroxymethyl) are major compounds found in methanol extract, and phenol, 2‐(1,1‐dimethylethyl)‐4‐(1,1,3,3‐tetramethylbutyl); 2,4‐di‐tert‐butylphenol; 2,4,6‐tris‐isopropylacetophenone are major compounds found in the chloroform extract. Table 5 shows the biological activity of the major chemical compounds. A major antioxidant and antibacterial component found in AP methanol extract is 2,3‐dihydro‐3,5‐dihydroxy‐6‐methyl, which is commonly known as DDMP (Ban et al. 2007). The reaction that occurs between reducing sugar carbonyl groups and amino groups in fruits, called the Maillard reaction, is responsible for the production of DDMP (Kim and Baltes 1996). The other important compound was 2,4a,5,5‐tetramethyl‐4a,5,6,7,8,8a‐hexahydro‐4H naphthalene‐1‐one, which is an antibacterial, antioxidant, and immunostimulant (Ponnamma and Manjunath 2012). Hexadecanoic acid, also known as palmitic acid, possesses mild antioxidant properties. The AP extract also included (ç) sitosterol, phytosterols present in many plants that have potent antiangiogenic, antifungal, and antibacterial properties (Zhang and Zhou 2011; Balamurugan et al. 2011). Another compound that also possesses anti‐mutagenic activity is 2‐furan carboxaldehyde 5 (hydroxymethyl)‐ (Abdulmalik et al. 2005). Vitamin C (ascorbic acid), phenol, 2,4‐di‐tert‐butylphenol, and 2‐(1,1‐dimethylethyl)‐4‐(1,1,3,3‐tetramethylbutyl) possess antioxidant, antimicrobial, nematicidal, and insecticidal activity (Glynn et al. 2007; Zhao et al. 2020).

**TABLE 3:** List of bioactive compounds identified in the methanol extract of apple pomace by GC/MS.

Compounds	RT	%Area	Molecular wt.	Molecular formula
Melezitose	17.57	26.52	504	C_18_H_32_O_16_
2,3‐dihydro‐3,5‐dihydroxy‐6‐methyl, 4H‐pyran‐4‐one	13.06	19.49	144	C_6_H_8_O_4_
6‐acetyl‐á‐d‐mannose	14.68	7.36	222	C_8_H_14_O_7_
5‐hydroxymethylfurfural	14.68	7.36	126	C_6_H_6_O_3_
*N*‐propargyloxycarbonyl‐, isohexyl ester, D‐Alanine	11.82	5.54	255	C_13_H_21_NO_4_
ç‐sitosterol	30.82	5.22	414	C_29_H_50_O
DL‐arabinose	6.1	4.74	150	C_5_H_10_O_5_
L‐ Glucose	15.14	3.4	180	C_6_H_12_O_6_
2‐thiophenecarboxylic acid,5‐nonyl—	15.43	2.74	254	C_14_H_22_O_2_S
Hexadecanoic acid, methyl ester	22.87	2.2	270	C_17_H_34_O_2_
Furan‐2‐carbohydrazide, N2‐(3‐indolylmethylene)—	16.89	2.18	253	C_14_H_11_N_3_O_2_
Maltose	15.34	2.06	342	C_12_H_22_O_11_
16‐octadecenoic acid, methyl ester	24.61	1.39	296	C_19_H_36_O_2_
Stigmasterol	24.09	1.37	412	C_29_H_48_O
Furfural	6.39	1.32	96	C_5_H_4_O_2_
Acetic acid,2‐(2‐pyrrolidinylideneamino)—	6.9	1.07	142	C_6_H_10_N_2_O_2_
Cyclohexane,1,4‐dimethyl‐2‐octadecyl—	9.33	0.54	364	C_26_H_52_
Lactose	18.33	0.45	342	C_12_H_22_O_11_
Ergosta‐5,22‐dien‐3‐ol, acetate, (3á,22E)—	24.22	0.28	440	C_30_H_48_O_2_

**TABLE 4:** List of bioactive compounds identified in apple pomace chloroform extract by GC–MS.

Compounds	RT	% Area	Molecular wt.	Molecular formula
2,4,6‐Tri‐isopropylacetophenone	24.72	36.94	246	C_17_H_26_O
Phenol, 2‐(1,1‐dimethylethyl)‐4‐(1,1,3,3‐t etramethylbutyl)—	25.89	20.67	262	C_18_H_30_O
1H‐Inden‐1‐one, 2,3‐dihydro‐5,6‐dimethoxy‐3‐methyl	22.88	13.24	206	C_12_H_14_O_3_
Heptaethylene glycol monododecyl ether	29.76	8.46	494	C_26_H_54_O_8_
15,15′‐Bi‐1,4,7,10,13‐pentaoxacyc lohexadecane	27.62	2.69	466	C_22_H_42_O_10_
(1S,17S)‐3,6,9,12,15,18,21,24,27,30‐Decaoxabicyclo[15.13.0]triacontane	26.84	2.39	438	C_20_H_38_O_10_
(2S, 2′S)‐2, 2′‐Bis[1, 4, 7, 10, 13‐pentaoxacyclopentadecane]	25.23	1.96	438	C_20_H_38_O_10_
2,4‐Di‐tert‐butylphenol	23.06	1.76	206	C_14_H_22_O
Octaethylene glycol monododecylethen	25.36	1.03	538	C_28_H_58_O_9_

**TABLE 5:** Biological activities of bioactive compounds identified in apple pomace methanol and chloroform extract by using GC–MS.

Compound name	Chemical class	Chemical structure	Biological activity	References
Cyclohexane, 1,4‐dimethyl‐2‐octadecyl—	Aldehydes	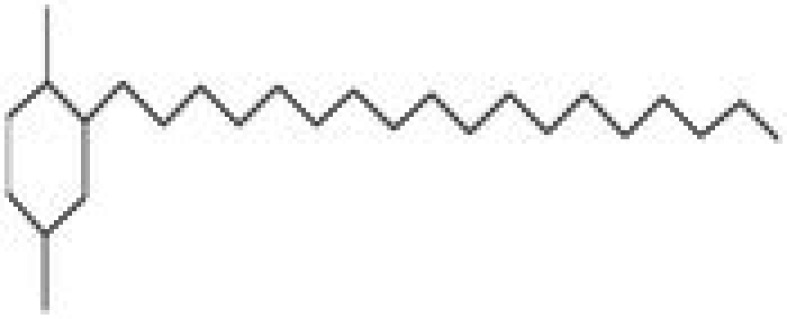	Antibacterial, antioxidant, antitumor, and anti‐cancer	Yu et al. (2013)
2,3‐dihydro‐3,5‐dihydroxy‐6‐methyl, 4H‐pyran‐4‐one	Ketones	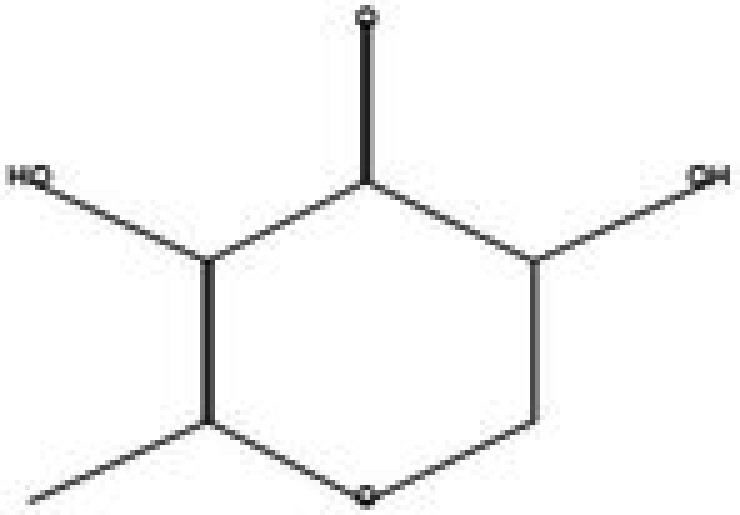	Antioxidant, anti‐inflammatory, antimicrobial, anti‐cancer, and anti‐diabetic	Banakar and Jayaraj (2018)
5‐hydroxymethylfurfural	Aromatic hydrocarbon	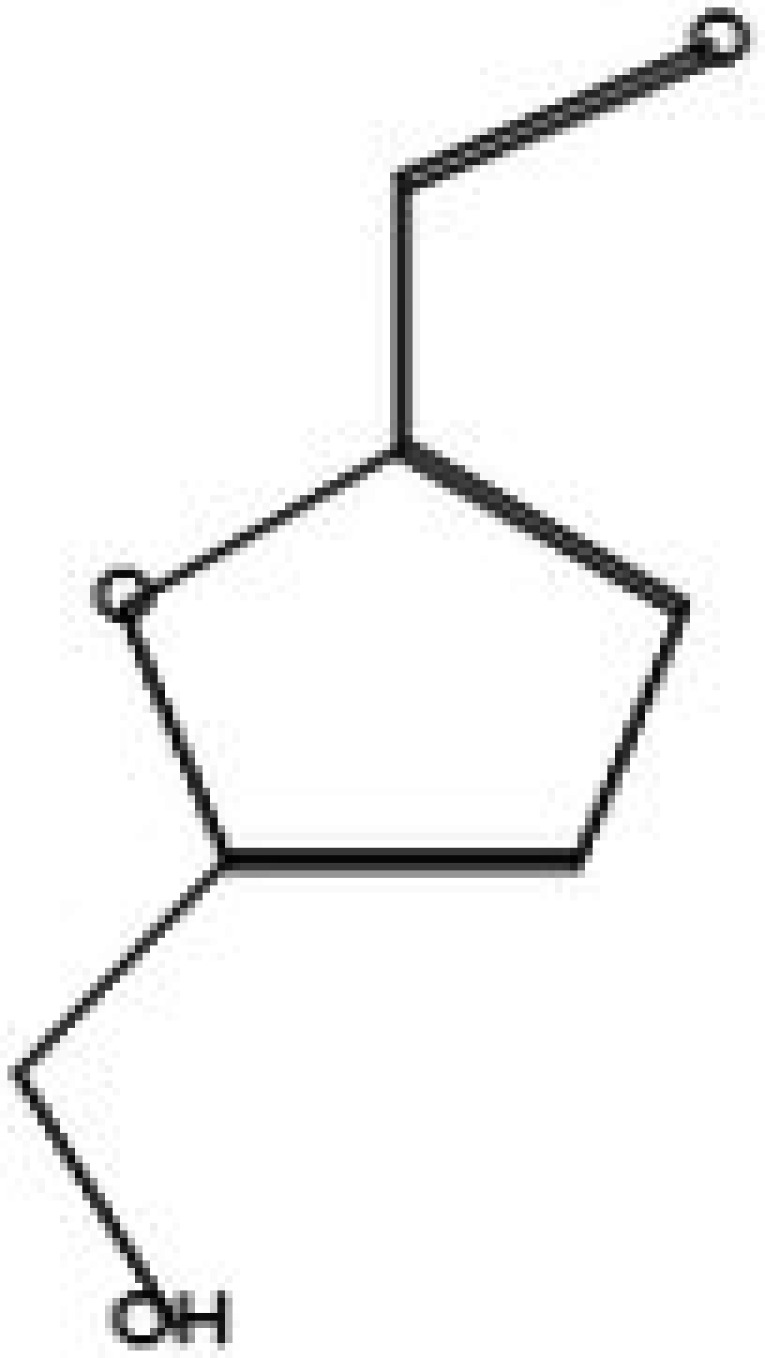	Antioxidant, antimicrobial	Yadav and Gupta (2015)
Furan‐2‐carbohydrazide, N2‐(3‐indolylmethylene)—	Alkenes	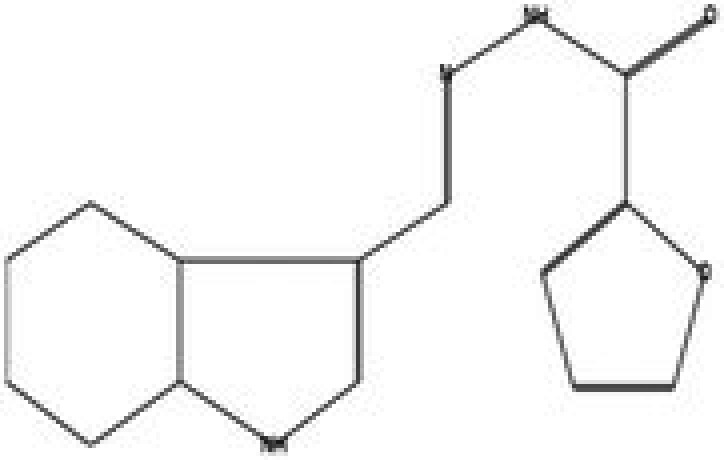	Antibacterial, antioxidant, immunostimulant, and anti‐cancer	Madrera and Valles (2011)
Hexadecanoic acid, methyl ester	Acid	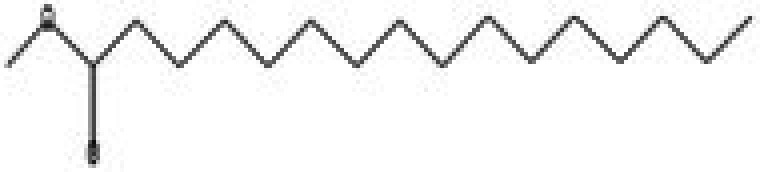	Antiatherosclerotic, antioxidant, and antibacterial	Rahman et al. (2013)
Ergosta‐5,22‐dien‐3‐ol, acetate, (3á,22E)—	Alcohol	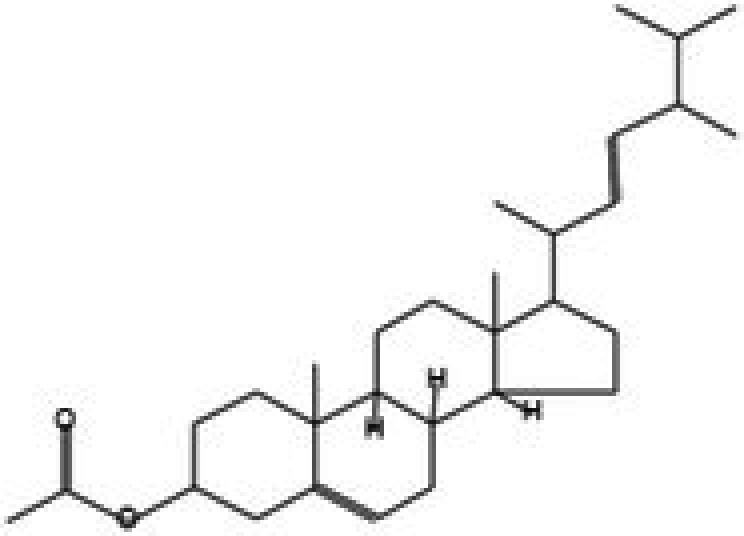	Anti‐inflammatory, anti‐cancer, and antimicrobial	Suleimen et al. (2021)
16‐octadecenoic acid, methylester	Ester	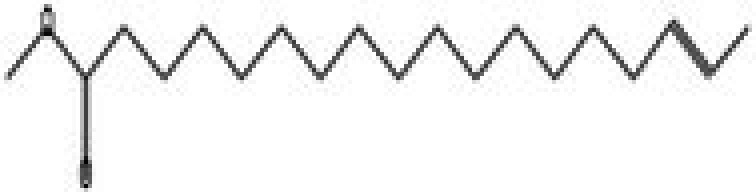	Anti‐inflammatory, antibacterial, hypocholesterolemic, and hepatoprotective	Mazumder et al. (2020)
ç‐sitosterol	Sterol	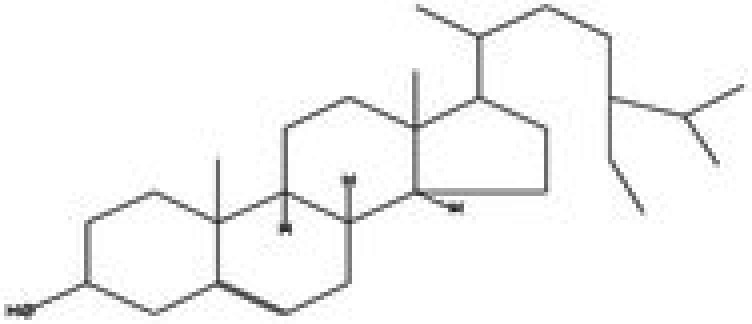	Antioxidant, immunomodulatory, anti‐diabetic, antimicrobial, anthelminthic, anti‐cancer, and anti‐mutagenic	Villaseñor et al. (2002)
2,4‐Di‐tert‐butylphenol or phenol	Phenylpropanes	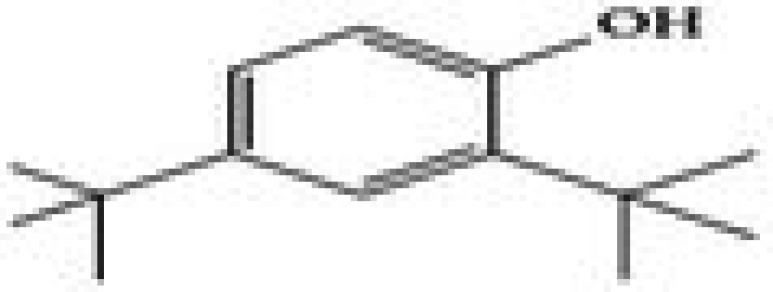	Antimicrobial, antioxidant, and anti‐inflammatory	CI and Indira (2016)

A previous study by Madrera and Valles (2011) reported 124 volatile compounds, including linalool and ethyl hexanoate, by using Stir Bar Sorptive Extraction (SBSE) combined with GC–MS. In another GC–MS analysis of AP, the presence of α‐tocopherol, 2, 3‐dihydro‐3, 5‐dihydroxy‐6‐methyl‐2 6, 10, 14, 18, 22, 4H‐Pyran‐4‐one, 2, 6, 10, 15, 19, 23‐hexamethyl‐, (all E)‐, α‐Sitosterol, Tetracosahexaene, 2‐Furancarboxaldehyde, 5(hydroxymethyl), and fatty acids has been observed (Madrera and Valles 2011).

Fărcaș et al. (2022) found that alcohols, esters, aldehydes, ketones, terpenes, acids, and other substances are among the 48 compounds that have been found and categorized. Esters were the most represented group, ranging between 44.81% and 53.29%, followed by ketones (4.14%–5.72%) and aldehydes (29.75%–43.99%). The most significant component in the alcohols category was 1‐hexanol, which ranged from 2.18% to 4.11%. In the ketones group, the principal representatives were acetophenone and 6‐methyl‐5‐hepten‐2‐one.

### 3.4 Antioxidant Activity of Apple Pomace

The antioxidant potential of AP was studied as mentioned in the methodology, and the results of this study are discussed in the following sections.

#### 3.4.1 Radical Scavenging Activity by RSA/DPPH and FRAP Assay

DPPH is commonly used to evaluate the potential of chemical compounds to act as free radical scavengers or hydrogen donors, as well as to calculate antioxidant activity in liquid and fruit samples. DPPH is a violet‐colored free radical that is sustained (Mantena et al. 2008; Pallauf et al. 2008). Table 6 depicts the findings of the DPPH and FRAP assay study of AP.

**TABLE 6:** Radical scavenging activity of apple pomace extracts.

Treatment groups	DPPH assay		FRAP assay
	(% RSA)	(IC_50_ Value)	Ferrous sulphate equivalents (μg FS/mg sample)
Methanol extract	65.72 ± 0.149b	332.69 ± 0.235b	34.57 ± 0.006b
Chloroform extract	49.85 ± 0.336 a	476.32 ± 0.375c	32.09 ± 0.006a
Ascorbic acid	77.66 ± 0.085 c	255.59 ± 0.048a	431.27 ± 0.020c

*Note:* All values are mean ± SD (*n* = 3). IC_50_: half‐minimum inhibitory concentration; different letters a, b, c means significant difference (*p* ≤ 0.05).

Abbreviation: RSA, radical scavenging activity.

After scavenging, free radicals change the color of DPPH from purple to pale yellow or colorless. The free radical scavenging activity of the methanol extract of AP was higher than chloroform but lower than ascorbic acid (positive control). The radial scavenging activities of methanol (65.72%) and chloroform (49.85%) extracts of AP in the present study were low (82.74%) as compared to the earlier findings by Rahman et al. (2013). The antioxidant ability of the extracts may also be analyzed using the IC_50_ values, which represent the quantity of extract necessary to reduce half of the free radicals present in the reaction mixture. Low antioxidant activity is shown by high IC_50_ values. The IC_50_ values of methanol (332.69 ± 0.235) and chloroform extracts (476.32 ± 0.375) were higher as compared to ascorbic acid (255.59 ± 0.048) in the present study (Table 6), which indicates ascorbic acid was the most potential radical scavenger, followed by methanol and chloroform extract.

The principle behind the Fe^3+^ reducing antioxidant power assay is the reduction of the ferric tripyridyl‐S‐triazine complex into a ferrous‐colored complex with the presence of antioxidants. As sample concentration increases, the antioxidants present in the samples reduce the ferric tripyridyl‐s‐triazine complex to produce a blue‐colored compound, increasing absorbance. Table 6 shows the ferrous sulfate equivalent of the AP methanol and chloroform extracts after FRAP assay. The present study results indicated that methanol extract (34.57 μg FS/mg) has higher antioxidant activity than chloroform extract (32.09 μg FS/mg). Rahman et al. (2013) observed the high antioxidant potential of AP due to high quantities of flavonoids and polyphenols as compared with the reducing activity of methanol extract (22.38 μg BE/mg). However, Grispoldi et al. (2022) reported a DPPH value of UAE of AP (11.77 ± 0.33 mg TE/g) which was higher than the FRAP value of UAE of AP 1.18 ± 0.01 mg TE/g and lower than the present study.

The FRAP activity was observed by Altemimi et al. (2023) as 64.43, 73.19, and 68.18 μg/mL for ethyl acetate, ethanol, and methanol extract of walnut ( *Juglans regia* L.) respectively, while DPPH radical scavenging activities of the same extracts were 47.66, 32.41, and 51.90 μg/mL, respectively.

### 3.5 Anti‐Bacterial Activity of Apple Pomace

The findings of the antibacterial activity of AP are shown in Table 7. The present study reported the anti‐microbial activity of AP extracts against the Gram‐negative bacteria *Klebsiella pneumoniae* (MTCC 109), *S. typhimurium* (MTCC 3858), and *E. coli* (MTCC 443), with ampicillin sodium salt used as a positive control. The minimum inhibitory concentration used for the sample was 62.5 μg/mL, and the maximum inhibitory concentration was 500 μg/mL. The anti‐bacterial activities of AP methanolic and AP chloroform extracts against Gram (−ve) bacteria, *E. coli* , *S. typhimurium*, and *K. pneumoniae* were in the range of 80–320 μg/mL and 160–640 μg/mL, respectively. The anti‐bacterial activity of AP against Gram (+ve) bacteria, *B. cereus* (MTCC 1305) and *E. faecalis* (MTCC 1032) was found to be 320 and 80 μg/mL for methanol extract and 160 and 40 μg/mL for chloroform extract, respectively. A study of the anti‐bacterial activity of walnuts by Altemimi et al. (2023) found 2–8 μg/mL against *Bacillus subtilis, Staphylococcus aureus, Pseudomonas aeruginosa, and E. coli*. The MICs of golden apple ethyl acetate extract against *E. coli* and *S. aureus* were 2500 and 1250 μg/mL, respectively, as observed by Zhang et al. (2016).

**TABLE 7:** Antibacterial assay of methanolic and chloroform extract of apple pomace.

Microbes used	Minimum inhibitory concentrations in μg/mL		
	Ampicillin sodium salt^a^ (μg/mL)	Methanolic AP extract (μg/mL)	Chloroform AP extract (μg/mL)
*Escherichia coli* MTCC 443	1.0	80	160
*Klebsiella pneumonia* MTCC *109*	0.25	160	320
*Salmonella typhimurium* MTCC 3858	1.0	320	640
*Bacillus cereus* MTCC 1305	1.0	320	160
*Enterococcus faecalis* MTCC 1032	0.50	80	40

^a^ Ampicillin sodium salt used as a standard.

### 3.6 Extraction of Soluble Sugars From AP

**FIGURE 2:**
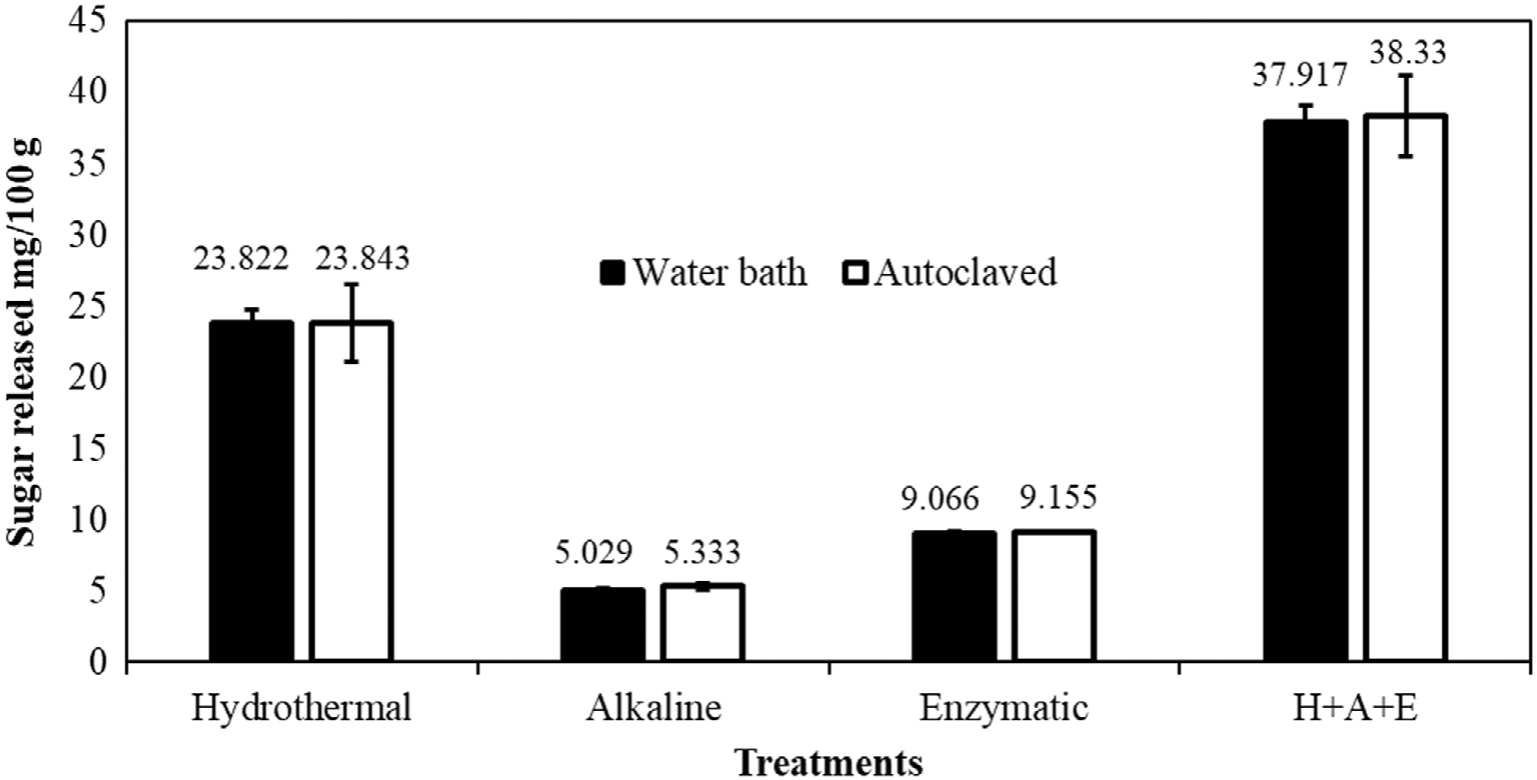
Reduced sugar extracted from apple pomace after pretreatment and hydrolysis with enzyme (H + A + E = hydrothermal + alkaline + enzymatic) under experimental conditions.

The results of hydrothermal treatment of AP at 100°C and 121°C are shown in Figure 2. After hydrothermal treatment at 100°C and 121°C, for 30 min the quantity of reducing sugar released was 23.82% and 23.84%, respectively, which was about two times the initial (12.22%) amount of reducing sugar.

After hydrothermal treatment of AP, the solid residue contained lipids, soluble dietary fiber, and a few mineral elements. The extracts were collected and used to obtain non‐polar substances. These substances were removed by alkaline (1% NaOH) pretreatment followed by enzymatic hydrolysis. In the present study, other than extracts, the solid residue had a significant concentration of complex carbohydrates, that is, lignin and cellulose. As a result, after hydrothermal and alkaline treatment, hydrolysis with enzymes is necessary to convert cellulose, lignin, and pectin (complex carbohydrates) into fermentable, soluble (hydrolyzed) sugars. Pectinase and cellulase enzymes were utilized in this investigation for enzymatic hydrolysis, and the results are presented in Figure 2. Hydrolysis of AP with enzymes was optimized using various parameters as mentioned in the methodology and after optimization of all the conditions, it was observed that the maximum (37.91 and 38.33 mg/100 g) release of reducing sugars after enzymatic hydrolysis of AP was reported with cellulase and pectinase (1 mg of enzymes/g of AP) at 50°C, pH 5.0, and 24 h of treatment. These conditions were selected for use in subsequent experiments. It was observed that the combination of hydrothermal and enzymatic hydrolysis released the maximum amount of reducing sugar as compared to hydrothermal and enzymatic hydrolysis alone (Figure 2).

Surya and Kumar (2018) observed that the combination of both treatments that yield the maximum amount of reducing sugars is 43.1 g/100 g AP, 10 times more than the reducing sugar present initially (4.1 g/100 g) in AP while fructose (27.2%, DW), sucrose (16.3%, DW), and glucose (8.0%, DW) were the main soluble sugars that were extracted from AP using 85% ethanol and trace quantities of xylose (0.2%, DW), galactose (0.3%, DW), and arabinose (0.1%, DW) reported by Jin et al. (2019).

## 4 Conclusion and Future Aspects

The present study tried to evaluate the chemical composition of AP as well as its hydrolysis for the recovery of soluble and insoluble sugars. The result shows that AP is a good source of nutritional components with nutraceutical properties. The fresh AP powder showed a low pH, which can help to prevent the growth of pathogenic microbes. Total dietary fiber has a larger quantitative composition than proteins and lipids, showing that AP is an excellent source of dietary food supplements. In terms of functional components, the data suggest that AP might be an appealing source of biologically active substances due to a large proportion of insoluble fibers compared to soluble fibers and significant quantities of ascorbic acid, polyphenols, and flavonoids. Some minerals, that is, Fe, K, Zn, Ca, and Mn, are also found in the highest amounts. The findings of this investigation indicate that the AP extract contains secondary metabolites, that is, acids, esters, and alcohols, and shows the presence of good antioxidant and anti‐bacterial potential. This study also reveals that enzymatic hydrolysis with hydrothermal treatment increases the availability of reducing sugars in AP and serves as a low‐cost substrate for sugar‐based biorefinery.

In addition to making a substantial contribution to the circular economy, AP may be converted into high‐value products that can boost industrial revenue sources without compromising human food security. Eco‐friendly technologies have proven to be a potential substitute for traditional methods to increase extraction yields, saving processing time, and reducing the environmental impact of harmful solvents. Despite this, industrial procedures that are in line with a circular economy still need to be developed. As a result, using the valorization concept facilitates the conversion of AP into valuable products with relevant potential uses in the pharmaceutical and human consumption sectors, such as the extraction of certain compounds and the production of innovative functional food items.

## Author Contributions

**Nisha Devi:** methodology, analysis, visualization, and writing an original draft. **Natália Cruz‐Martins, Dinesh Kumar,** and **Vinod Kumar:** methodology, visualization, and editing of the manuscript. **Tabarak Malik:** funding acquisition and supervision. **Gurvendra Pal Singh, Rachna Verma,** and **Muhammad Torequl Islam:** visualization and editing of the manuscript.

## Conflicts of Interest

The authors declare no conflicts of interest.

## Data Availability

The data that support the findings of this study are available on request from the corresponding author.
